# Atrophic Postacne Scar Treatment: Narrative Review

**DOI:** 10.2196/49954

**Published:** 2024-02-21

**Authors:** Enas Attia

**Affiliations:** 1 Department of Dermatology, Venereology, and Andrology, Faculty of Medicine Ain Shams University Cairo Egypt; 2 Department of Dermatology Ain Al Khaleej Hospital Abu Dhabi United Arab Emirates

**Keywords:** acne, atrophic scars, treatment, acne scarring, scars, scarring, well-being, psychosocial well-being, psychosocial, physical well-being, self-esteem, face, facial scarring, implications, skin, dermatology, dermatologist

## Abstract

Acne scarring is a frequent complication of acne. Scars negatively impact psychosocial and physical well‐being. Optimal treatments significantly improve the appearance, quality of life, and self-esteem of people with scarring. A wide range of interventions have been proposed for acne scars. This narrative review aimed to focus on facial atrophic scarring interventions. The management of acne scarring includes various types of resurfacing (chemical peels, lasers, and dermabrasion); the use of injectable fillers; and surgical methods, such as needling, punch excision, punch elevation, or subcision. Since the scarred tissue has impaired regeneration abilities, the future implementation of stem or progenitor regenerative medical techniques is likely to add considerable value. There are limited randomized controlled trials that aimed to determine which treatment options should be considered the gold standard. Combining interventions would likely produce more benefit compared to the implementation of a single method.

## Introduction

Atrophic scars present clinically as indentations in the skin due to destructive inflammation in the deep dermis as a result of delayed or inadequate acne treatment. Atrophic postacne scars are further classified into ice-pick scars (V‐shaped epithelial tracts with a sharp margin that can extend deeper in the skin), boxcar scars (a round-to-oval scar with sharp vertical sides that can extend deeper in the skin), and rolling scars (irregular scars with a rolling or undulating shape) [1]. Atrophic postacne scar risk assessment depends on the worst-ever severity of acne, the duration of acne, family history of atrophic postacne scars, and lesion manipulation behaviors. This provides a dichotomous outcome: lower versus higher risk of developing scars [2].

Early effective treatment of acne is the best strategy to prevent or limit postacne scarring. Different factors influence the treatment choice for acne scars, for example, color, texture, distensibility, and morphology. For example, the selection of the chemical peeling agent and concentration depends on the patient’s skin type and severity of scarring. Moreover, considering the flexibility and low cost, chemical peels, in general, play an important role in the management of all grades of acne scars. However, trichloroacetic acid (TCA) chemical peeling carries the risk of postinflammatory hyperpigmentation (PIH), particularly in darker skin phototypes [3]. Regarding lasers, choosing the type and appropriate settings while taking into consideration the depth of the scar, skin type, and tendency to develop PIH is of utmost importance [4]. Nevertheless, severe scars are poorly treated and do not improve greatly with resurfacing procedures, where punch excision and punch elevation can be tried instead [3].

Preprocedure considerations include the acne-free period, isotretinoin-free period, history of skin infections (eg, herpes virus), history of general or local skin disorders affecting healing, history of keloids or hypertrophic scarring, history of tanning, skin phototype, and sun exposure habits, as well as history of systemic or local therapies affecting healing [5]. The management of acne scarring includes various types of resurfacing (chemical peels, lasers, and dermabrasion); the use of injectable fillers; and surgical methods, such as needling, subcision, punch excision, or punch elevation [6].

This narrative review aimed to focus on facial atrophic scarring interventions in brief. The outcomes, including adverse events, participant satisfaction, and postprocedure downtime, are reviewed.

## Methods

A PubMed literature review was conducted, and the search keywords included a combination of the following keywords: “acne,” “scars,” and “treatment.” The synonyms “management,” “modalities,” and “therapy” were also considered, along with the names of different modalities such as “laser,” “radiofrequency,” “needling, microneedle, micro needling or microneedling,” “dermaroller,” “dermabrasion, microdermabrasion or micro dermabrasion,” “chemical peel, chemical peeling or chemical peels,” “platelet rich plasma,” “stem cells,” “fillers,” “subcision,” “punch,” “growth factor,” “ozone,” and “botulinum toxin.”

The articles regarding clinical trials, meta-analyses, and systematic reviews with at least an English abstract that were published before June 1, 2023, were included.

Articles discussing interventions for nonfacial or other types of scars were excluded.

## Results

### Scars-Associated Erythema Management

Treating scars-associated erythema (SAE) can be an initial and dramatic step toward improving acne scarring. Pulsed dye laser (PDL) is the gold standard. It uses selective thermolysis to destroy vascular components of the dermis, leading to clinical improvement of erythema. The major chromophore is oxyhemoglobin, which absorbs light in the yellow and green range, with peaks at 418, 542, and 577 nm. The long-pulsed PDL (595-600 nm) slowly heats target vessels with less risk of postprocedure purpura. In addition to treating SAE, PDL also induces collagen remodeling, thus improving the depressed appearance of scars [7].

Other laser and light devices include the potassium titanyl phosphate laser, also known as the frequency-doubled neodymium-doped yttrium aluminum garnet (Nd:YAG) laser; 1550-nm erbium-doped fractional laser (EDL); and intense pulsed light (IPL) [8]. The use of the potassium titanyl phosphate laser leads to significant improvement in the vascular component without significant effects on collagen remodeling [9].

In addition to being a frontline agent for atrophic scars, the 1550-nm wavelength emitted by EDL penetrates approximately 1000 μm into the skin to target tissue water, allowing for the improvement of erythema through microvascular destruction of vessels deeper in the dermis [10].

IPL does not typically produce purpura, and larger spot sizes allow for a greater surface area to be treated deeper and more quickly. However, given the range of wavelengths that may be used; adjacent, competing chromophore absorption peaks; and poor specificity, drawing conclusions regarding efficacy in treating SAE with IPL is difficult. Moreover, care must also be taken to avoid postinflammatory hypopigmentation and PIH in darker skin phototypes [7].

### Ablative Laser Resurfacing

Traditional ablative laser resurfacing removes the epidermis and part of the dermis of the scars, allowing collagen remodeling and re‐epithelialization. Ablative 10,600-nm carbon dioxide (CO_2_) lasers and 2940-nm erbium-doped yttrium aluminum garnet (Er:YAG) lasers are the most commonly used ablative lasers for acne scars. CO_2_ lasers cause denaturation and thermal stimulus in the tissues surrounding ablation, promoting wound healing and the production of myofibroblasts and matrix proteins [11]. Adverse effects include persistent erythema, hypopigmentation, PIH, infection, scarring, and a relatively long recovery period (weeks) [12].

Fractional laser resurfacing acts, as the name indicates, on regularly spaced arrays over a fraction of the skin surface to induce thermal ablation of microscopic columns of epidermal and dermal tissue. Microscopic columns of light or microthermal zones (MTZs) leave the intervening skin unaffected and minimize damage to the epidermis. The skin adjacent to sites of laser injury remains intact, allowing for rapid postprocedural re-epithelialization due to the migration of intact cells into the damaged microcolumns [13]. This approach provides a faster recovery when compared with conventional ablative resurfacing [14].

Fractional 10,600-nm CO_2_ laser; 2940-nm Er:YAG laser; 2790-nm erbium-doped yttrium scandium gallium garnet laser; 1540-nm erbium glass (Er:glass) laser; and 1550-nm EDL produce comparable rates of improvement in atrophic acne scars after multiple treatments. The least responsive scar type is ice-pick scars [7]. Adverse effects include erythema that lasts for days to weeks, PIH that lasts for weeks, and procedural discomfort. These lasers are safer in darker skin phototypes, with less dyschromia than ablative lasers. Lower densities have been associated with less risk for hyperpigmentation [15]. The deeper penetration of the laser might lead to contraction of the underlying muscle, so lower energy and densities should be used on the periocular region [7]. Fractional 1540‐nm Er:glass laser treatment for 3 sessions at 4‐week intervals improved scar texture and severity [16].

### Nonablative Laser Resurfacing

Nonablative laser resurfacing, such as the short- and long-pulsed and Q-switched Nd:YAG lasers and diode lasers, produces dermal thermal injury while preserving the epidermis; this promotes collagen remodeling, which leads to improvement in scarring [17]. Results are accordingly modest (20%-30%), and multiple treatment sessions are required to achieve typically less impressive results. Postprocedure side effects are minimal, with erythema lasting less than 2 hours and no reports of pain, swelling, oozing, or scarring. Using the 532-nm Nd:YAG laser for an average of 3 treatments improved scars by an average of 53.6%, with a range from 10% to 90% [18]. The use of the nonfractional, nonablative Q-switched 1064-nm Nd:YAG laser (4 sessions at 4‐week intervals) resulted in a more than 50% improvement in 3 out of 32 patients with acne scarring [19].

The picosecond 755-nm Alexandrite laser delivers shorter pulse durations with lower fluences of energy and, therefore, leads to fewer adverse effects. With the aid of a diffractive lens array, which delivers pulses 500 μm apart, it permits the treatment of a greater surface area, improving the appearance and texture of atrophic rolling scars similar to fractional ablative lasers. This technology has a favorable safety profile for darker skin phototypes; the mean pain score is mild; and downtime is minimal, with transient erythema and edema and no exfoliation, vesiculation, crusting, scarring, hypopigmentation, or PIH [20].

### Radiofrequency

Nonablative radiofrequency (RF) treatments deliver a current through the dermis that stimulates dermal remodeling. With traditional unipolar or monopolar RF, a single electrode allows for penetration deep into the dermis, but this is associated with increased pain and discomfort [21]. Bipolar RF allows for the delivery of a more focused current to the dermis. Fractional RF uses an array of electrodes to create zones of thermal wounds that stimulate dermal remodeling. Microneedles can be used to deliver RF to a particular depth within the dermis. Microneedle bipolar RF and fractional RF treatments offer the best results for acne scarring, particularly ice-pick and boxcar scars [22]. Needling and ablative fractional lasers are tolerable and safe procedures with no significant difference in the treatment of skin scars in 60% of previous studies [23]. The adverse reactions associated with RF include transient pain, erythema, and scabbing that resolve within days [7]. Zhang et al [24] found that fractional RF sessions resulted in comparable improvement of acne scars after fractional lasers, with no PIH observed on the areas treated with fractional RF.

### Skin Needling

Skin needling procedures may diminish the appearance of acne scars. A needling device is rolled over the surface of the skin to form numerous perforations in the epidermis and dermis, with the goal of stimulating new collagen [25]. The advantages of skin needling include low cost, a relatively short recovery period (2-3 days), and a very low risk for PIH [26].

Skin needling treatment is well tolerated by most people and the pain is minimal. The full result may take 8 to 12 months as the deposition of new collagen takes place slowly [25].

One important advantage is that the epidermis remains intact, eliminating most of the risks of chemical peeling or laser resurfacing. Furthermore, microneedling provides a clear channel for the efficient absorption of topical agents, including platelet-rich plasma (PRP), which can improve cosmetic results [27].

### Dermabrasion and Microdermabrasion

Dermabrasion involves the use of tools (eg, high‐speed brush, diamond cylinder, fraise, or silicon carbide sandpaper) to remove the epidermis or the epidermis and part of the dermis. An advantage of the procedure is that it allows the clinician to target scar edges precisely without thermal injury. It may be effective for some acne scars but is usually not used for ice-pick or deep boxcar scars. Adverse effects include significant pain, a considerable recovery time, scarring, pigment alterations, and milia formation [28].

Microdermabrasion (MDA) is a minimally invasive epidermal resurfacing procedure, in which abrasive crystals are propelled against the skin under the control of a handheld vacuum system. The crystals cause gentle mechanical abrasion to the skin, which ultimately removes the stratum corneum layer of the epidermis. As part of the wound healing process, new epidermis forms with enhanced cosmesis [29]. Half-side comparison between combined MDA plus aminolevulinic acid–photodynamic therapy (PDT) versus combined MDA plus placebo‐PDT for 5 sessions (4‐week intervals) showed more improvement of scarring on the combined MDA plus aminolevulinic acid–PDT split‐face than the combined MDA plus placebo‐PDT split‐face using the Physician’s Global Assessment of Acne Scarring scale [30].

### Chemical Peels

Chemical peels (using glycolic acid, lactic acid, salicylic acid, mandelic acid, TCA, or phenol) are used in treating small, depressed scars but not ice-pick or deep boxcar scars [31,32]. They induce injury to the skin that stimulates collagen remodeling and are categorized as superficial, medium, and deep based on the depth of the injury [7].

Superficial peels, such as lactic acid, salicylic acid, glycolic acid, Jessner solution, and 10% to 20% TCA, only affect the epidermis. Medium depth peels, such as combined Jessner solution with 25% to 35% TCA, affect the epidermis and papillary dermis. Deep peels, such as 50% or higher TCA and phenol (carbolic acid), injure skin to the midreticular dermis. Complications, including prolonged erythema, infection, PIH, and scarring, are more common in darker skin phototypes, deeper peels, and sun exposure. Phenol has been associated with cardiac toxicity related to systemic absorption [7].

Serial biweekly application of glycolic acid peels with different concentrations in a gradually increasing manner (2‐week intervals) is better than 15% glycolic acid cream applied daily for 24 weeks [33]. The chemical reconstruction of skin scars (CROSS) chemical peeling method applied twice every 12 weeks had comparable results to the use of the 1550-nm Er:glass fractional laser for 3 sessions (6‐week intervals) [34]. Four sessions (4‐week intervals) of chemical peeling using full‐strength TCA (100% TCA) CROSS showed equivalent improvement as 4 sessions (4‐week intervals) of skin needling using a dermaroller, with reported transient PIH in the peeling group [35]. Six sessions (4 weeks apart) of chemical peeling with 20% TCA combined with skin needling showed comparable improvement as 6 sessions (4 weeks apart) of fractional nonablative 1540-nm Er:glass laser treatment, with more than 50% improvement in acne scars [36]. Ultrapulsed CO_2_ fractional laser combined with 30% supramolecular salicylic acid has better efficacy in the treatment of acne scars than laser alone, and according to patient self-assessment, the combined treatment has a greater degree of improvement in acne scars and does not increase patient pain scores and related adverse reactions [37]. Four sessions (6‐week intervals) of chemical peeling with 20% TCA combined with skin needling is superior to deep peeling using a non–hydro‐alcoholic solution of oil phenol in 60% concentration formula [38].

### PRP and Stem Cell Therapy

Autologous PRP can enhance wound healing by accelerating tissue repair through the release of growth factors, cytokines, and chemokines from their granules. Intradermal injections of PRP were first noted to improve acne scarring when used for skin rejuvenation. Topical PRP has a synergistic effect with skin needling in atrophic acne scars, as skin needling creates a way for PRP absorption and allows platelets to contribute to wound healing. PRP as both an intradermal injection and topical application in fluid or gel form after fractional ablative CO_2_ laser therapy enhanced the recovery of laser-damaged skin and improved the clinical appearance of acne scars [39-41].

Mesenchymal stem cells (MSCs) are capable of differentiation into various cell lineages and have been shown to promote wound healing [42]. MSCs can be isolated from umbilical cord blood and expanded [43]. In contrast to umbilical cord MSCs, adipose tissue–derived MSCs are relatively easy to obtain. One injection of autologous adipose tissue–derived adult stem cells is as effective as 3 sessions of fractional CO_2_ laser in the treatment of atrophic acne scars [44].

### Filler

Injectable fillers have been proposed to improve the appearance of atrophic acne scars, including collagen, autologous fat transfer, and artificial injectable fillers [45]. Hyaluronic acid (HA) fillers typically last for a few months, making repeated treatments necessary, which increases cost [7]. Semipermanent fillers can last up to 2 years and are biostimulatory; they include poly-ι-lactic acid and calcium hydroxylapatite [46,47]. Permanent fillers comprise larger particles that cannot be phagocytosed. They can last from several years to lifelong but can be displaced over time due to changes in the adjacent connective tissue. Silicone is relatively cheap and is stable for 10 to 20 years. Polymethylmethacrylate is a synthetic permanent filler suspended in bovine collagen and lidocaine [7]. Solomon et al [48] injected 96 patients with acne scars with polymethylmethacrylate, resulting in 99.0% improvement, high patient satisfaction, and a good safety profile.

O’Daniel [49] implemented an individualized multimodal approach in patients with atrophic acne scars and aging. Resurfacing techniques were used to correct surface irregularities, long-lasting dermal fillers were used addressed the volume loss resulting from acne scars, and subsuperficial musculoaponeurotic system face-lift procedures were used to counter the soft tissue laxity and ptosis associated with aging. In the author’s clinical practice, multimodal approaches incorporating fractionated laser, injectable poly-L: -lactic acid, and subsuperficial musculoaponeurotic system face-lift procedures have achieved optimal aesthetic outcomes, high patient satisfaction, and durability of aesthetic effect over time.

Autologous fat grafting, PRP, and stromal vascular fraction are effective and safe for the treatment of acne scars. Autologous fat grafting and stromal vascular fraction may be a better treatment for acne scars than PRP. However, this hypothesis still needs to be tested in the future in large randomized controlled trials [50].

### Individual Atrophic Scars Surgical Management

Punch excision may be an effective treatment for ice-pick scars and small (<3 mm) boxcar scars. A punch biopsy instrument of equal or slightly greater diameter than the scar is used to incise the tissue to the subcutaneous fat layer and excise the scar. Some authors espouse punch excision followed by secondary intention healing, in which a scar is created but is less noticeable because of change at the depth of the base. It has been associated with good results, but secondary widening of the scar may occur [28]. The defect should be closed by sutures along relaxed skin tension lines. Placing a single nonabsorbable suture for punch holes 2.5 mm or larger might facilitate wound healing and minimize spreading [7]. For scars larger than 3.5 mm, elliptical excision may be more favorable than punch excision [51].

Punch elevation is best suited for boxcar scars. The scar border is excised, leaving the deepest part of the scar that is adherent to the fat layer. The scar is raised higher than the surrounding skin; it then retracts during healing to become level with the surface [28].

Fractional CO_2_ laser preceded by punch elevation produced a more than 50% improvement in acne scars after 2 sessions [52].

Subcision is used for the management of rolling or depressed scars; a blade inserted parallel to the skin surface is used to cut fibrotic strands tethering the scar to the underlying tissue [53]. Reported adverse effects include bruising, swelling, bleeding, and infection [54]. RF-assisted subcision was found to be comparable to convention subcision with no risk of hematoma, but entry point burn can occur [55]. Using microplasma RF technology combined with subcision to treat depressed scars obtained relatively satisfactory results with no adverse effects [56].

It is of note that blunt cannula subcision is more effective than Nokor needle subcision for acne scars treatment [57]. Injectable fillers showed comparable results to 18‐gauge Nokor needle subcision [58], yet bruising from subcision was significantly worse than that from injection, whereas lumpiness from fillers was significantly worse than that from subcision. Significant and persistent improvement of acne scars, without considerable complications, was noted after the combined protocol of subcision, followed by HA filler initially, and then followed by fractional CO_2_ laser 2 weeks later [59]. Subcision combined with HA or threads could offer a more significant, clinical improvement of acne scars than subcision alone [60].

Subcision with autologous fat grafting showed better yet nonsignificant results versus subcision with PRP injection in the treatment of postacne scars [61]. However, one study comparing subcision with PRP injection versus normal saline showed similar efficacy, denoting that subcision, similar to the mechanical effect of injecting solution, is more important than the nature of the solution in the treatment of atrophic acne scars [62].

### Other Treatments

Treatment with topical epidermal growth factor after ablative fractional CO_2_ laser is safe and improves the clinical appearance of atrophic acne scars. Epidermal growth factor may help decrease skin pigmentation after laser treatment [63].

Botulinum toxin type A microtoxin, when injected intradermally as microdroplets, can be used to reduce pore size, sebum production, rosacea, acne, scars, and fine lines. Intradermal injection can also be used for the safe prevention and management of scars [64].

Ozone has been gaining greater visibility for its possible antioxidant effects when used in human dermatological pathologies, including skin scarring. However, more studies with better methodological standards and longer-term assessments of side effects should be conducted to achieve better standards and safety in ozone therapy for dermatological conditions [65].

The main treatments for atrophic postacne scars discussed in this review are summarized in Table 1.

**Table 1:** Procedures for atrophic postacne scars.

Procedure and techniques			Advantages		Disadvantages
**Vascular lasers or light**					
	PDL^a^, KTP^b^, EDL^c^, and IPL^d^	Improve SAE^e^ and may induce collagen remodeling		PIH^f^	
**Ablative lasers**					
	Ablative CO_2_^g^ and Er:YAG^h^	Remove epidermis and part of the dermis, allowing collagen remodeling and re‐epithelialization		Persistent erythema, hypopigmentation, PIH, infection, scarring, and long recovery period	
**Fractional ablative lasers**					
	Fractional CO_2_, 2940-nm Er:YAG, 2790-nm Er:YSGG^i^, 1540-nm Er:glass^j^, and 1550-nm EDL	Faster recovery, safer in darker skin phototypes, and less dyschromia		Poor results for ice-pick scars, erythema, PIH, and procedural discomfort	
**Nonablative lasers**					
	Q-switched Nd:YAG^k^, diode, and picosecond 755-nm Alexandrite	Dermal thermal injury while preserving epidermis; minimal side effects: short erythema and minimal pain, swelling, oozing, scarring, or downtime		Results are modest and less impressive	
**RF^l^**					
	Fractional RF +/– needling	Create zones of thermal wounds to stimulate dermal remodeling; microneedle bipolar RF and fractional RF offer the best results for ice-pick and boxcar scars with no PIH		Transient pain, erythema, and scabbing	
**Needling**					
	Needling device rolled over skin	Low cost, well tolerated, increase transepidermal absorption of topical agents, short recovery period, and low PIH		The full result may take 8 to 12 months as the deposition of new collagen takes place slowly	
**Dermabrasion and microdermabrasion**					
	High‐speed brush, diamond cylinder, fraise, silicon carbide sandpaper, and abrasive crystals	Mechanical resurfacing procedures target scar edges precisely without thermal injury		Not effective for ice-pick or deep boxcar scars	
**Chemical peels**					
	Glycolic acid, lactic acid, salicylic acid, mandelic acid, TCA^m^, and phenol	Induce chemical injury to the skin that stimulates collagen remodeling		Prolonged erythema, infection, PIH, and scarring in darker skin phototypes, deeper peels, and sun exposure; phenol has cardiac toxicity related to systemic absorption	
**PRP and stem cell therapy**					
	Autologous PRP^n^, MSCs^o^, and adipose tissue–derived MSCs	Enhance wound healing through the release of growth factors, cytokines, and chemokines		Better when combined with skin needling or fractional laser	
**Filler**					
	HA^p^ fillers, PLL^q^, and CaHA^r^	Address the volume loss resulting from atrophic acne scars		Lumpiness and temporary results, making repeated treatments necessary, which increases cost	
**Individual atrophic scars surgical management**					
	Punch excision	Suitable for ice-pick scars and small (<3 mm) boxcar scars +/– sutures along relaxed skin tension lines		Secondary widening of the scar may occur	
	Elliptical excision	More favorable than punch excision in larger scars		Secondary scar may occur	
	Punch elevation	For boxcar scars		Better when followed by fractional CO_2_ laser	
	Subcision	A blade is used to cut fibrotic strands tethering the scar		Bruising, swelling, bleeding, and infection	
	RF-assisted subcision	Comparable to convention subcision with no hematoma		Entry point burn	
	Microplasma RF technology combined with subcision	Satisfactory results with relatively no adverse effects		Short-term pain, edema, erythema, scaling, and effusion	

^a^PDL: pulsed dye laser.

^b^KTP: potassium titanyl phosphate.

^c^EDL: erbium-doped fractional laser.

^d^IPL: intense pulsed light.

^e^SAE: scars-associated erythema.

^f^PIH: postinflammatory hyperpigmentation.

^g^CO_2_: carbon dioxide.

^h^Er:YAG: erbium-doped yttrium aluminum garnet.

^i^Er:YSGG: erbium-doped yttrium scandium gallium garnet.

^j^Er:glass: erbium glass.

^k^Nd:YAG: neodymium-doped yttrium aluminum garnet.

^l^RF: radiofrequency.

^m^TCA: trichloroacetic acid.

^n^PRP: platelet-rich plasma.

^o^MSC: mesenchymal stem cell.

^p^HA: hyaluronic acid.

^q^PLL: poly-ι-lactic acid.

^r^CaHA: calcium hydroxylapatite.

## Discussion

### Principal Findings

Acne scarring is a frequent complication of acne. Early effective treatment of acne is the best strategy to prevent or limit postacne scarring. Treating SAE is the gold-standard, initial, and dramatic step toward improving acne scarring.

The management of acne scarring includes various types of resurfacing (chemical peels, lasers, and dermabrasion); the use of injectable fillers; and surgical methods, such as needling, punch excision, punch elevation, or subcision. There are limited randomized controlled trials that aimed to determine which treatment should be considered the gold standard.

Less invasive, less traumatizing procedures are more appreciated with less side effects and less downtime. Injectable fillers improve atrophic acne scars; however, the impermanence of their effect and their minimal utility for fine, shallow, and sharply depressed scars should be also considered.

The *Energy-Based Devices for the Treatment of Acne Scars: 2022 International Consensus Recommendations* considered energy-based devices to be a first-line treatment for a variety of acne scar types and stated that patients without access to these treatments may not be receiving the best available care for optimal cosmetic results [66]. The consensus recommended future high-quality research and updated international treatment guidelines and reimbursement schemes to reflect this status.

Combining interventions likely produce more benefit compared with the implementation of a single method. Since the scarred tissue has impaired regeneration abilities, the future implementation of stem or progenitor regenerative medical techniques is likely to add considerable value. One readily available strategy is PRP, which appears to be a safe and effective treatment for various types of atrophic scars. In addition, when added to ablative lasers or microneedling, it seems to considerably add to the efficacy of treatment and reduce the side effects [67]. Platelet-rich fibrin (PRF), a second-generation platelet concentrate, was developed for the purpose of overcoming the limitations of PRP. PRF can produce a higher cumulative release of growth factors than PRP. The therapeutic response was significantly higher in PRF than PRP either alone or combined with needling [68].

### Conclusions

Early effective treatment of acne is the best strategy to prevent or limit postacne scarring. Treating SAE is the gold-standard, initial, and dramatic step toward improving acne scarring. Combining less invasive, less traumatizing procedures is more beneficial and more appreciated with less side effects and less downtime.

Future studies should recruit sufficient participants for blinded trials and include combined therapies versus placebo. Trials should collect baseline variables (participant demographics, acne lesions and extent, skin phototype, scar duration, and depth of scars) to ensure that they are balanced. Trials outcomes should be assessed by both participants and investigators, including adverse events, participant satisfaction, and quality of life, as well as cost and postprocedure downtime.

## Abbreviations

CO2: carbon dioxide
CROSS: chemical reconstruction of skin scars
EDL: erbium-doped fractional laser
Er glass: erbium glass
Er YAG: erbium-doped yttrium aluminum garnet
HA: hyaluronic acid
IPL: intense pulsed light
MDA: microdermabrasion
MSC: mesenchymal stem cell
MTZ: microthermal zone
Nd YAG: neodymium-doped yttrium aluminum garnet
PDL: pulsed dye laser
PDT: photodynamic therapy
PIH: postinflammatory hyperpigmentation
PRF: platelet-rich fibrin
PRP: platelet-rich plasma
RF: radiofrequency
SAE: scars-associated erythema
TCA: trichloroacetic acid
